# The Prevalence and Pattern of Superficial Fungal Infections among School Children in Ile-Ife, South-Western Nigeria

**DOI:** 10.1155/2014/842917

**Published:** 2014-12-10

**Authors:** Olaide Olutoyin Oke, Olaniyi Onayemi, Olayinka Abimbola Olasode, Akinlolu Gabriel Omisore, Olumayowa Abimbola Oninla

**Affiliations:** ^1^Dermatology Unit, Department of Internal Medicine, Federal Medical Centre, Abeokuta 110222, Nigeria; ^2^Department of Dermatology & Venereology, Obafemi Awolowo University, Ile-Ife, Nigeria; ^3^Department of Community Medicine, Osun State University, Osogbo, Nigeria

## Abstract

Fungal infections of the skin and nails are common global problems with attendant morbidity among affected individuals. Children are mostly affected due to predisposing factors such as overcrowding and low socioeconomic factors. The aim of this study was to determine the prevalence and the clinical patterns of superficial fungal infections among primary school children in Ile-Ife. A multistage sampling was conducted to select eight hundred pupils from ten primary schools in Ile-Ife. Data on epidemiological characteristics and clinical history was collected using a semistructured questionnaire and skin scrapings were done. The prevalence of superficial fungal infections among the 800 respondents was 35.0%. Male pupils constituted 51.0% of respondents while the females were 49.0%. The mean age for all the respondents was 9.42 ± 2.00. Tinea capitis was the commonest infection with a prevalence of 26.9% and tinea unguium, tinea corporis, and tinea faciei had a prevalence of 0.8%, 0.6%, and 0.5%, respectively. Tinea manuum had the least prevalence of 0.1%. Pityriasis versicolor had a prevalence of 4.4%. *Microsporum audouinii* was the leading organism isolated. The study shows that the prevalence of superficial fungal infection (SFI) among primary school children in Ile-Ife is high with tinea capitis as the commonest SFI.

## 1. Introduction

Fungal infections of the skin and nails have been found in the last few decades to affect 20–25% of the world's population, making them one of the most frequent forms of infection [1]. They represent a major public health problem in school-age children especially in low- and middle-income countries (LMICs) like Nigeria where possible predisposing factors to acquiring the infection such as hygiene, overcrowding, and low socioeconomic factors remain present [2]. Prevalence of superficial fungal infection (SFI) in Nigeria as reported ranges from 3.4% to 55% [3–6].

The superficial fungal infections include those caused by dermatophytes (such as tinea capitis, tinea faciei, tinea corporis, tinea unguium, tinea manuum, and tinea pedis) and nondermatophytes such as pityriasis versicolor, cutaneous candidiasis, tinea nigra, black piedra, and white piedra [2]. Tinea capitis is the commonest superficial fungal infection among primary school children [1]. In developing countries including Africa,* Microsporum audouinii* and* Trichophyton soudanense *are most frequently isolated aetiological agents whereas this has been displaced by* Microsporum canis* and* Trichophyton tonsurans* in most European countries [3]. The most common and most widely distributed aetiological agent is* Trichophyton rubrum*, which causes different types of infection in different parts of the world [1].

Many studies have been done on SFI in Nigeria; however, most of them are limited and are not recent; therefore, a more recent and comprehensive study is needed to assess the impact of this problem. The quality of life has been shown to be impaired in children with tinea capitis [5]. It has also been documented that prevalence and aetiological agents vary from time to time with geographic zone, age, humidity, and sex [3]; thus it becomes imperative not only to know the prevalence of SFI but also to ascertain the pattern of SFI, not only in terms of sociodemographic characteristics but also in terms of the specific causative organisms. Thus, this study was designed to determine the current prevalence and pattern of SFI in an urban area of Nigeria, and thus can provide additional information on the trends of SFI in Nigeria. Findings from this study will provide up-to-date information on SFI for evidence-based action aimed at reducing the morbidity of the infection.

## 2. Subjects, Materials, and Methods

This cross sectional study was conducted between January and March 2011 in Ile-Ife, Osun State, South-Western Nigeria. Ile-Ife is an ancient city that is believed to be the source of the Yoruba people.

A multistage sampling technique was used that involved selection along local governments, schools (both public and private), classes, and proportionate pupils from selected class strata.

A total of 800 school children were recruited from 10 schools—6 publicly funded and 4 privately owned primary schools in Ile-Ife, Nigeria. Calculated minimum sample size using prevalence from a previous study [5] was 255 but this was increased to 800. The purpose and benefits of the study were explained to the pupils, their parents/guardians, teachers, and head-teachers. Only pupils whose parents/guardians gave informed consent were eventually included in the study.

Ethical clearance was obtained from the Ethical Committee, Obafemi Awolowo University Teaching Hospitals Complex, Ile-Ife, Osun State, Nigeria.

Quantitative data was collected from pupils in selected schools using an interviewer administered questionnaire. The semistructured precoded questionnaire had sections focusing on the sociodemographic details of the respondents, possible predisposing factors for developing superficial fungal infections, and socioeconomic status of parents. Physical examination was conducted in a well-lit room and the pupils were examined thoroughly from head to toe with minimal clothing for the presence of any superficial fungal infection. The fungal infections were then classified.

Diagnosis was made clinically, and appropriate skin scrapings or nail clippings were taken to confirm diagnosis. Pupils who had superficial fungal infections were treated appropriately by the authors.

### 2.1. Sample Collection and Processing

Areas of the skin suspected to have fungal infections were scraped using disposable scalpel blades after first cleaning with alcohol. The scrapings were collected on a sterile brown paper and transported to the laboratory within 2 hours for microscopic and culture analysis. The scrapings were handled separately ensuring that no individual scrapings mixed. Nail clippings were also done as required. For direct microscopy, each specimen was placed on a slide and a drop of 10% potassium hydroxide added before covering with a cover slip. This was then heated gently (for about five minutes) to soften it and it was then examined for the presence of hyphae and/or arthroconidia under low (X10) and high (X40) power objective.

Diagnosis of dermatophytes in hair pieces was made by the visualisation of arthroconidia arranged along the length of the hair in chains or masses around the hair (ectothrix infection) or in the hair substance (endothrix infection). The scrapings and the pieces of hair were plated out separately on Sabouraud's dextrose agar. Cycloheximide was employed because saprophytic fungi and yeasts normally present as contaminants will be inhibited by it. Chloramphenicol and streptomycin were the antibiotics used to inhibit bacterial contaminants. Culture plates were incubated at 27°C for 4 weeks and then examined for the presence of dermatophytes. Macro- and micromorphological studies of cultured colonies were done for the presence of dermatophytes.

### 2.2. Data Analysis

The data obtained were entered and analysed using Statistical Package for Social Sciences version 16.0 (SPSS, IBM Corporation, Armonk, NY, USA). Some of the variables were regrouped and/or recoded before the data analysis was done. Continuous data were expressed as means ± standard deviation (SD) and categorical data as percentages. Differences between categorical variables were analyzed using chi-square test. The level of statistical significance for all the tests was a *P* value <0.05.

## 3. Results

### 3.1. General Characteristics/Prevalence

A total of 800 pupils were recruited for the study. The mean age for all the respondents was 9.42 ± 2.00. Males made up 51% (408 pupils) of the respondents while the females were 49% (392 pupils).

Out of the 800 pupils, 280 pupils were found to have superficial fungal infection (SFI) giving a prevalence of 35%. Highest prevalence was found among the age group 9–12 years. Males were more affected than the females as it occurred in 40.6% and 29.1% of them, respectively, and this difference was statistically significant. Similarly, more respondents from the Hausa ethnic group had SFI compared to other tribes (Table 1).

### 3.2. Clinical Types of SFI Seen

**Figure 1:**
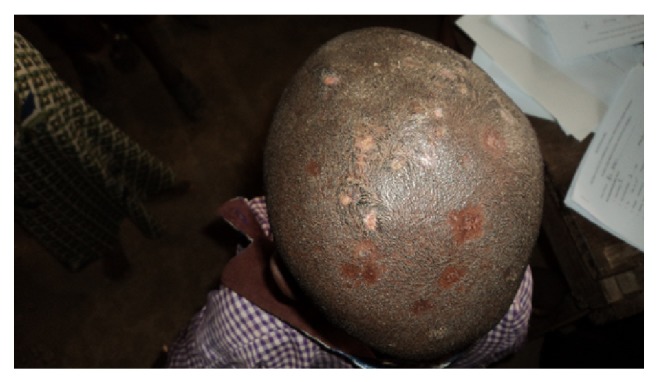
Tinea capitis with scarring alopecia.

**Figure 2:**
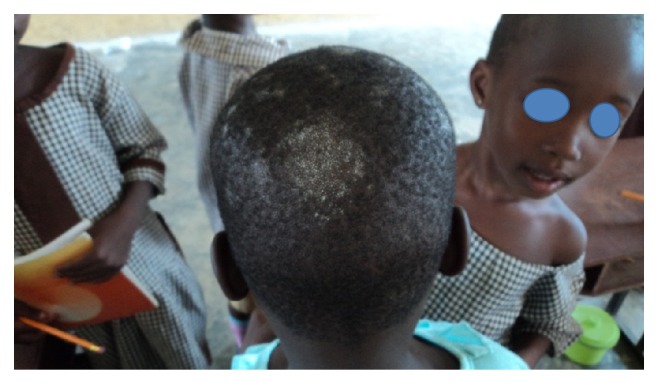
Tinea capitis.

**Figure 3:**
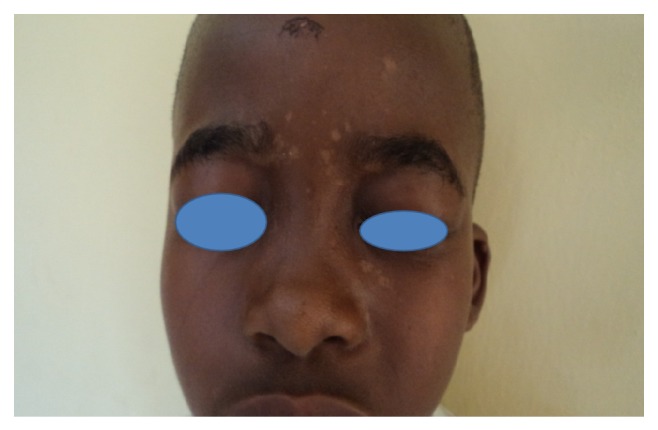
Pityriasis versicolor on the face.

**Figure 4:**
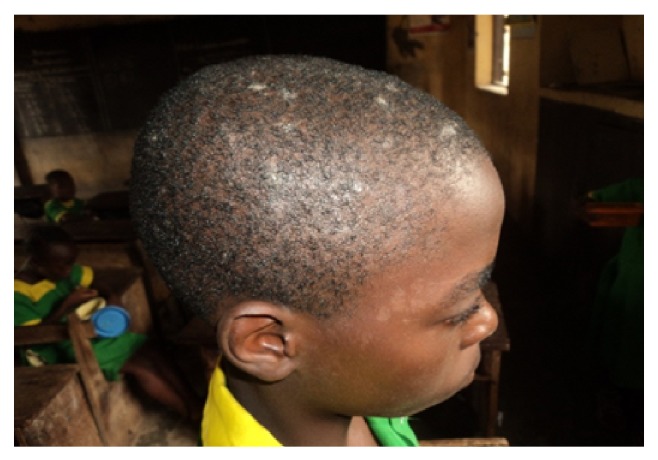
Tinea capitis and pityriasis versicolor.

**Figure 5:**
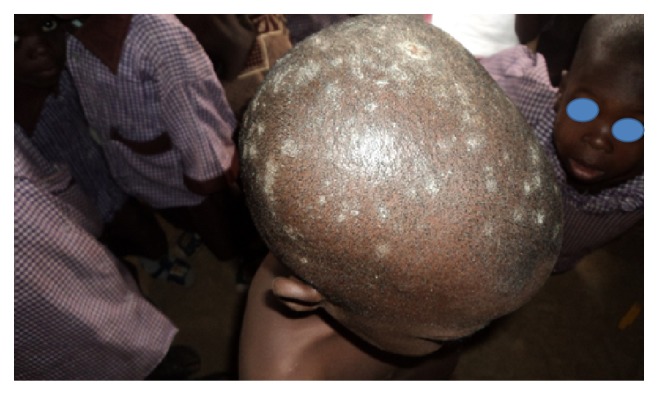
Tinea capitis.

**Figure 6:**
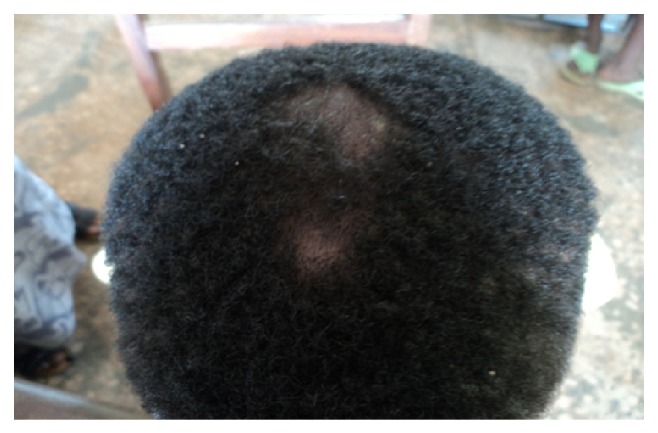
Tinea capitis with alopecia.

**Figure 7:**
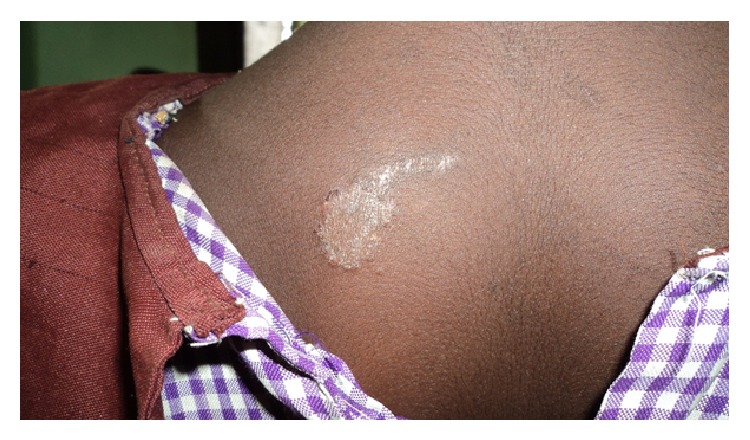
Tinea corporis.

**Figure 8:**
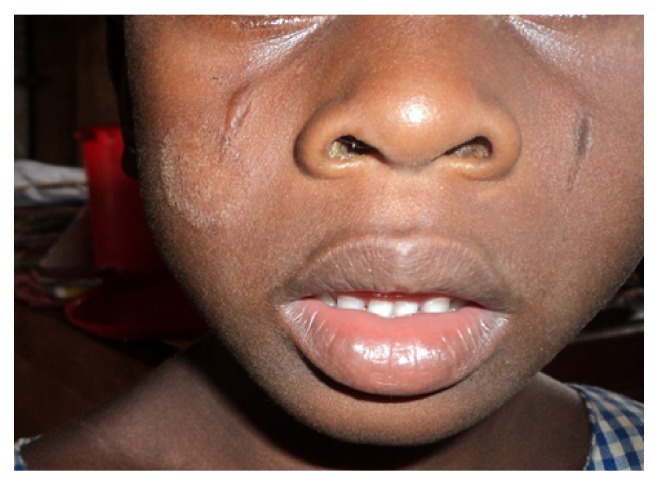
Tinea faciei.

In terms of clinical types, tinea capitis was the commonest accounting for 26.9% of the prevalence. Tinea unguium, tinea corporis, and tinea faciei had a prevalence of 0.8%, 0.6%, and 0.5%, respectively. Tinea manuum had the least prevalence of 0.1%. Pityriasis versicolor, a nondermatophytosis was seen with a prevalence of 4.4%. In some pupils, more than one type of superficial fungal infection was seen. Tinea capitis with pityriasis versicolor was seen in 12 cases (1.5%); tinea capitis with tinea unguium and tinea capitis with tinea faciei were seen in 0.1% of total prevalence, respectively. There was no case of tinea pedis or any form of candidiasis seen in the course of the study (Table 2) (see Figures 1, 2, 3, 4, 5, 6, 7, and 8).

### 3.3. Clinical Pattern of SFI Seen

Two hundred and nine pupils (95%) with tinea capitis, either singly or in combination had noninflammatory form with the grey patch type accounting for 46.3%. This was followed by the black dot type and then seborrheic dermatitis-like tinea capitis that constituted 27.1% and 17.9%, respectively. As regards the inflammatory form, the pustular type was the most common (6.5%) of all types of tinea capitis seen, while 2.2% of the pupils were found to have kerion. There was no case of favus seen among all the pupils that were examined. The back was the commonest site (60%) of tinea corporis, while the face was the commonest site (95.7%) for pityriasis versicolor. Tinea unguium involving the fingernails was seen in 85.7% of cases while involvement of the toenails occurred in the remaining 14.3% (Table 3).

### 3.4. Characteristics of Aetiological Agents Isolated

The distribution of the species isolated from the pupils with clinically suspected superficial fungal infections (according to age, gender, and clinical types) is as shown in Tables 4(a) and 4(b).

Of the 800 pupils recruited for the study, 280 pupils were found to have lesions with clinical suspicion of superficial fungal infection. 35 of the pupils did not give consent to have their skin or nail scraped. Of the 245 pupils that had skin scrapings or nail clippings done for suspected superficial fungal infection, 157 (64.1%) samples were mycologically proven and 88 (35.9%) were culture negative.

Dermatophytes constituted 72.7% of the samples (first five species in Table 4) while nondermatophyte molds were 27.3%. Concerning the dermatophytes, the 3 genera,* Microsporum*,* Trichophyton*, and* Epidermophyton*, were represented with 5 different species that included* Microsporum audouinii* as the leading organism isolated (28%). This was followed by* Trichophyton rubrum* (21.7%).* Epidermophyton floccosum* was the least isolated (5.1%). Other isolates in the study were* Trichophyton mentagrophytes* and* Trichophyton schoenleinii*. The nondermatophyte molds identified were* Penicillium* (12%),* Aspergillus fumigatus* (8.4%), and* Aspergillus niger* (6.4%).

About half of the isolates of the dermatophytes were from pupils aged 4–6 years and 7–11 years (49% and 49.7%, resp.). Only 1.3% were from the age group 12–16 years.

The frequency distribution of the positive cases among the males and females was statistically significant with positive isolates being more among males than females (Table 4).

## 4. Discussion

### 4.1. Prevalence of Superficial Fungal Infection

Superficial fungal infections are common and remain an important public health problem among children worldwide and particularly in Nigeria [5–7]. This is evident in this study where prevalence of superficial fungal infection was 35%. This prevalence is comparably slightly higher than the 21% observed in a study in Ebonyi State, South-Eastern Nigeria [4]. Similar studies in Iraq and Egypt had a low prevalence of 2.7% and 7.4%, respectively [8]. It has been suggested that differences in the prevalence of superficial fungal infection in different regions may be due to variation in climatic and environmental conditions of the areas being studied [5, 9].

Tinea capitis, a dermatophytosis, was the predominant superficial fungal infection and this corroborates other studies that showed that tinea capitis is the commonest superficial fungal infection among children [6, 9–11]. Reasons that can explain the predominance of tinea capitis among children of primary school age include use of local barbers, poor personal hygiene, short hair that promotes transmission from one scalp to the other, and increased frequent contacts with playmates at school and younger siblings at home [12, 13].

The noninflammatory form of tinea capitis was the commoner tinea capitis lesion and out of this the grey patch type was the most common. This is comparable to other studies [11, 14, 15]. Kerion has been observed to occur more in males compared to females and this was also observed in this study [5]. Tinea capitis was also found in association with tinea faciei, tinea unguium, and pityriasis versicolor in some pupils. This is possible as the dermatophytes can spread from the scalp to other regions through autoinoculation.

Pityriasis versicolor which was the next common type of superficial fungal infection is a nondermatophytosis. Its prevalence of 4.4% is similar to that found in Ibadan, South-Western Nigeria, and in Abakaliki, South-Eastern Nigeria, where the prevalence was 4.6% and 4.7%, respectively [10, 11], and in Taiwan where they also recorded a prevalence of 4.4%, but comparatively higher than what was obtained in Bamako, Mali (1.6%) [11]. Other dermatophytoses such as tinea unguium, tinea corporis, tinea faciei, and tinea manuum all had a prevalence that was <1%. This is corroborated by previous studies that show these infections to be uncommon in the age group being studied as they are not usually exposed to the predisposing factors such as involvement in gardening which predispose them to tinea manuum [16, 17].

There was no case of tinea pedis or candidiasis found in this study. This is probably due to the age group that is being studied. Tinea pedis has been found more with older age group as documented by Moon et al. [18]. It is also believed that the older age groups are likely to wear their shoes more constantly and this with the hot and humid environment promoting moisture will favour susceptibility to the infection [12].

### 4.2. Sociodemographic Characteristics and the Presence of Superficial Fungal Infections

This study showed that superficial fungal infection was commoner in males (40.7%) than females, in a ratio of 1.4 : 1. Males usually keep short hair and visit the barbers' shop more, have more frequent contacts with playmates, play with sand more, and are less concerned about hygiene and personal grooming than the females [12]. Also females usually weave their hair and visit the barbers less [12]. This male predominance was also observed in previous studies done both within and outside Nigeria [1, 9, 19].

Superficial fungal infection has a higher prevalence amongst children less than twelve years of age. This is comparable to what Nweze obtained in North-Eastern Nigeria and in other studies [3, 20]. Tinea capitis in particular was higher in children less than seven years of age. Presence of superficial fungal infection in younger age group observed in this study supports the suggestion that infection is related to the poor hygiene at the younger age and absence of saturated fatty acids that provide a natural protective mechanism against fungal infections [21]. Furthermore, Ayanbimpe et al. attributed the highest rate of infection amongst them to the fact that they are the group active at playgrounds and will thus have closer contact with sources of pathogens [19].

### 4.3. Characteristics of Aetiological Agents Identified

The dermatophyte species isolated in this study belonged to the three genera* Trichophyton*,* Microsporum*, and* Epidermophyton* of which five species, mostly anthropophilic, were identified.* Microsporum audouinii* was the commonest specie followed by* Trichophyton rubrum*. Others included* T. mentagrophytes*,* T. schoenleinii,* and* Epidermophyton floccosum*. The nondermatophyte mould identified were* Aspergillus fumigatus*,* Aspergillus niger*, and* Penicillium*. Most of the species were more predominant in the males. Studies have established variability in the species of dermatophytes isolated from one geographical region to the other and also per time [19]. In Nigeria, Nweze documented that the spectrum of pathogens and their clinical presentations in West Africa are different from those seen in other continents [22]. This finding was corroborated in this study.

*Microsporum audouinii* was the commonest dermatophyte species isolated (28%) and this is similar to findings done by Soyinka decades ago and also by Ajao and Akintunde both in Ile-Ife (the study site) on superficial fungal infections and tinea capitis, respectively, and by Enweani et al. at Ekpoma located in a different geopolitical zone from Ile-Ife [6, 12, 23]. In other parts of the country, it seems* Microsporum audouinii* has ceased to be the dominant aetiological agent where* Trichophyton soudanese*,* Trichophyton mentagrophytes*, and other species predominate [17, 19, 24].* Microsporum audouinii* is mainly a human pathogen but occasionally it infects animals and has been observed to resolve as pupils approach puberty [5, 6, 12]; thus, in this study, it was only found among the age group 4–11 years.

*Trichophyton* species (*rubrum*,* mentagrophytes*, and* schoenleinii*) were isolated from 39.6% pupils with* Trichophyton rubrum* being the dominant species in 21.7% and these were the fungi most recovered among pupils with tinea unguium, tinea corporis, and tinea faciei. This is similar to findings obtained elsewhere [17, 25].

*Epidermophyton floccosum* was the least isolated (5.1%) and this is not unusual as it is usually isolated more in tinea pedis and there was no case of this fungal infection in the study [12].

Presence of nondermatophyte molds such as* Aspergillus *and* Penicillium *is becoming increasingly common and one of the factors attributed is the ubiquitous nature of their spores in our environment that makes it to be carried transiently on healthy skin [25, 26].

## 5. Conclusion

The prevalence of superficial fungal infection among primary school children in Ile-Ife, South-Western Nigeria, remains high. Tinea capitis was the most common fungal infection and the noninflammatory clinical type was most prevalent among them.* Microsporum audouinii* was the commonest organism isolated.

Regular health education about fungal infections that highlights their morbidities and modes of spread, should be given to school children, their parents and teachers, in order to truly reduce the prevalence and burden of superficial fungal infections in low and middle income countries (LMICs) such as Nigeria. Establishment of school-based dermatological services for primary school pupils will also be of immense help.

## Figures and Tables

**Table 1:** Relationship between the sociodemographic characteristics and the presence of superficial fungal infections.

	Presence of superficial fungal infections	Fungal Infections	Total *n* = 800	*χ* ^2^	df	*P* value
	Yes *n* = 280 (%)	No *n* = 520 (%)				
Age in years						
5–8	94 (31.97)	199 (67.9)	293	2.936	2	0.230
9–12	164 (37.61)	274 (62.6)	438			
13–16	22 (31.43)	47 (68.1)	69			
Sex						
Male	166 (40.7)	242 (59.3)	408	12.293	1	0.001
Female	114 (29.1)	278 (70.9)	392			
Class						
Pry 1–3	140 (34.3)	268 (65.7)	408	0.026	1	0.678
Pry 4–6	140 (35.7)	252 (64.3)	392			
Religion						
Christianity	214 (34.6)	404 (65.4)	618	0.662	2	0.586
Islam	66 (36.5)	115 (63.5)	181			
Traditional	0 (0)	1 (1.0)	1			
Ethnicity						
Yoruba	265 (35.7)	477	742	11.097	3	0.011
Hausa	7 (50.0)	7	14			
Igbo	2 (8.3)	22	24			
Others	6 (30.0)	14	20			

**Table 2:** Prevalence and Clinical types of superficial fungal infections among primary school children in Ile-Ife.

Type	Frequency	% Prevalence among all children examined (*n* = 800)	% Prevalence among those with infections (*n* = 280)
Tinea capitis alone	215	26.9	76.8
Pityriasis versicolor	35	4.4	12.5
Pityriasis versicolor and tinea capitis	12	1.5	4.3
Tinea unguium	6	0.8	2.1
Tinea corporis	5	0.6	1.8
Tinea faciei	4	0.5	1.4
Tinea manuum	1	0.1	0.4
Tinea capitis and tinea faciei	1	0.1	0.4
Tinea capitis and tinea unguium	1	0.1	0.4
Total	**280**	** 35**	**100.0**

**Table 3:** Clinical Patterns of SFI seen.

Tinea capitis	Number (%)
Noninflammatory types	
Grey patch type	106 (46.3)
Black dot type	62 (27.1)
Seborrheic dermatitis-like	41 (17.9
Inflammatory types	
Pustular	15 (6.5)
Kerion	5 (2.2)
Favus	0 (0)
Tinea corporis site	
Abdomen	1 (20)
Back	3 (60)
Limbs	1 (20)
Tinea unguium site	
Right middle finger	3 (42.8)
Right fourth finger	2 (28.6)
Left fourth and fifth fingers	1 (14.3)
Left great toe	1 (14.3)
Pityriasis versicolor site	
Face	45 (95.7)
Chest	2 (4.3)

**(a):** 

Species	Isolates according to age group				Isolates according to gender	
	Total (%)	4–6 yrs	7–11 yrs	12–16 yrs	Male (%)	Female (%)
*Microsporum audouinii *	44 (28.0)	13 (30.0)	31 (70.0)	0 (0.0)	32 (72.3)	12 (27.7)
*Trichophyton rubrum *	34 (21.7)	19 (55.9)	14 (41.2)	1 (2.9)	20 (58.8)	14 (41.2)
*Trichophyton mentagrophytes *	18 (11.5)	14 (77.8)	4 (22.2)	0 (0.0)	12 (66.7)	6 (33.3)
*Trichophyton schoenleinii *	10 (6.4)	8 (80.0)	2 (20.0)	0 (0.0)	7 (70.0)	3 (30.0)
*Epidermophyton floccosum *	8 (5.1)	2 (25.0)	6 (75.0)	0 (0.0)	4 (50.0)	4 (50.0)
*Aspergillus fumigatus *	14 (8.9)	8 (57.1)	6 (42.9)	0 (0.0)	11 (78.6)	3 (21.4)
*Aspergillus niger *	10 (6.4)	5 (50.0)	5 (50.0)	0 (0.0)	6 (60.0)	4 (40.0)
*Penicillium *	19 (12.0)	8 (42.1)	10 (52.6)	1 (5.3)	17 (89.5)	2 (10.5)
Total number (%)	**157** (**100.0**)	**77** (**49.0**)	**78** (**49.7**)	**2** (**1.3**)	**109** (**69.4**)	**48** (**30.6**)

^*^yrs—years.

**(b):** 

Clinical type	Isolate according to clinical type *n* = 157							
	*Microsporum audouinii* (%)	*Trichophyton rubrum* (%)	*Trichophyton mentagrophytes* (%)	*Trichophyton schoenleinii (%) *	*Epidermophyton floccosum* (%)	*Aspergillus fumigatus* (%)	*Aspergillus niger* (%)	*Penicillium* (%)
Tinea capitis	44 (100.0)	25 (73.5)	17 (94.4)	10 (100.0)	—	14 (100.0)	10 (100.0)	18 (94.7)
Tinea faciei	—	2 (5.9)	1 (5.6)	—	2 (25.0)	—	—	
Tinea corporis	—	4 (11.8)	—	—	1 (12.5)	—	—	1 (5.3)
Tinea manuum	—	1 (2.9)	—	—	—	—	—	
Tinea unguium	—	2 (5.9)	—	—	5 (62.5)	—	—	
Total	**44**	**34**	**18**	**10**	**8**	**14**	**10**	**19**
